# 4,4′-Bipyridine–2,2′-(1,2-phenylene­dioxy)diacetic acid–water (1/1/1)

**DOI:** 10.1107/S1600536810013905

**Published:** 2010-04-21

**Authors:** Feng-Quan Bu, Ying Wu, Ling Ye, Peng Yan

**Affiliations:** a School of Pharmaceutical Science, Jilin University, Changchun 130012, People’s Republic of China; b State Key Laboratory for Supramolecular Structure and Materials, Jilin University, Changchun 130012, People’s Republic of China; c Department of Chemistry and Chemical Engineering, Jinzhong College, Jinzhong 030600, People’s Republic of China

## Abstract

In the title 1:1:1 adduct, C_10_H_8_N_2_·C_10_H_10_O_6_·H_2_O, the dihedral angle between the rings of the 4,4-bipyridine molecule is 10.981 (8)°. In the crystal, O—H⋯O and O—H⋯N hydrogen bonds link the mol­ecules into a zigzag chain structure.

## Related literature

For the synthesis of 1,2-phenyl­enedi(oxyacetic acid), see: Mirci (1990 ▶). For related structures, see: Soleimannejad *et al.* (2009 ▶); Yu *et al.* (2006 ▶). For hydrogen-bonding motifs, see: Etter *et al.* (1990 ▶); Bernstein *et al.* (1995 ▶).
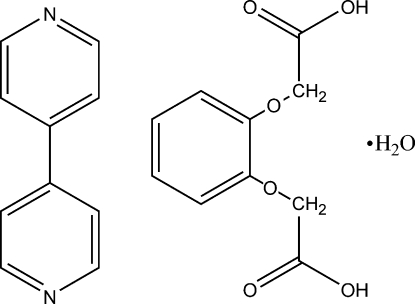


## Experimental

### 

#### Crystal data

C_10_H_8_N_2_·C_10_H_10_O_6_·H_2_O
*M* *_r_* = 400.38
Monoclinic, 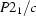

*a* = 11.566 (5) Å
*b* = 9.712 (5) Å
*c* = 16.810 (7) Å
β = 90.81 (2)°
*V* = 1888.2 (15) Å^3^
*Z* = 4
Mo *K*α radiation
μ = 0.11 mm^−1^
*T* = 291 K
0.20 × 0.18 × 0.12 mm

#### Data collection

Rigaku R-AXIS RAPID diffractometer
Absorption correction: multi-scan (*ABSCOR*; Higashi, 1995 ▶) *T*_min_ = 0.979, *T*_max_ = 0.987
18105 measured reflections
4306 independent reflections
2654 reflections with *I* > 2σ(*I*)
*R*_int_ = 0.030

#### Refinement

*R*[*F* ^2^ > 2σ(*F* ^2^)] = 0.039
*wR*(*F* ^2^) = 0.113
*S* = 0.97
4306 reflections
262 parameters
H-atom parameters constrained
Δρ_max_ = 0.17 e Å^−3^
Δρ_min_ = −0.15 e Å^−3^

### 

Data collection: *RAPID-AUTO* (Rigaku, 1998 ▶); cell refinement: *RAPID-AUTO*; data reduction: *CrystalClear* (Rigaku/MSC, 2002 ▶); program(s) used to solve structure: *SHELXS97* (Sheldrick, 2008 ▶); program(s) used to refine structure: *SHELXL97* (Sheldrick, 2008 ▶); molecular graphics: *ORTEPIII* (Burnett & Johnson, 1996 ▶), *ORTEP-3 for Windows* (Farrugia, 1997 ▶) and *SHELXTL* (Sheldrick, 2008 ▶); software used to prepare material for publication: *SHELXL97*.

## Supplementary Material

Crystal structure: contains datablocks I, global. DOI: 10.1107/S1600536810013905/dn2556sup1.cif

Structure factors: contains datablocks I. DOI: 10.1107/S1600536810013905/dn2556Isup2.hkl

Additional supplementary materials:  crystallographic information; 3D view; checkCIF report

## Figures and Tables

**Table 1:** Hydrogen-bond geometry (Å, °)

*D*—H⋯*A*	*D*—H	H⋯*A*	*D*⋯*A*	*D*—H⋯*A*
O3—H23⋯N2^i^	0.85	1.76	2.6051 (19)	173
O6—H24⋯N1	0.85	1.74	2.5871 (19)	176
O7—H21⋯O5	0.85	2.07	2.8817 (19)	160
O7—H22⋯O2	0.85	1.97	2.7826 (17)	161

Symmetry code: (i) 
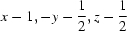
.

**Fig. 1.**
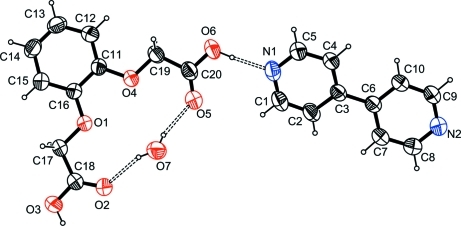
The molecular structure of the title compound with the atom labeling scheme, Ellipsoids are drawn at the 50% probability level. H atoms are represented as small spheres of arbitrary radii. Hydrogen bonds are shown as dashed lines.

**Fig. 2.**
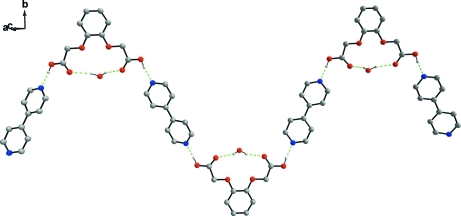
A partial packing view, showing the three-dimensional hydrogen bonding supramolecular network. Hydrogen bonds are shown as dashed lines.H atoms not involved in hydrogen bondings have been omitted for clarity.

## supplementary crystallographic information

### Comment

Flexible carboxylic acid and 4,4'-bipyridine are ofen used for constructing the high dimensional structure with intriguing topology. In this paper, we report the title compound, contained flexible 1,2-phenylenedi(oxyacetic acid), 4,4'-bipyridine and water molecules, which forms a one-dimensional hydrogen bonding chain strucutre.

The O—H···O hydrogen bonds link the 1,2-phenylenedi(oxyacetic acid) and water molecules into a 13-membered hydrogen bonding ring, with a unitary graph-set descriptorN1 = R^2^_2_(13) (Etter *et al.*, 1990; Bernstein *et al.*, 1995) (Figure 1, Table 1). And then, 4,4'-bipyridine molecules bridge these hydrogen bonding rings into a one-dimensional zigzag chain through the O—H···N hydrogen bonds (Table 1, Figure 2). Similar hydrogen bonding interactions involving the 4,4'-bipyridine have being frequently observed (Soleimannejad *et al.*, 2009; Yu *et al.*, 2006).

### Experimental

1,2-phenylenedi(oxyacetic acid) was prepared by the reaction of chloroacetic acid with odihydroxybenzene (Mirci, 1990 ). All other chemicals were analytical grade reagents and used without further purification. 1,2-phenylenedi (oxyacetic acid) (0.40 g, 2 mmol) and 4,4'-bipyridine (0.31 g, 2 mmol) were dissolved in mixed solution of water (5 ml) and ethanol (5 ml). Colorless rod crystals of title compound were obtained after several days in room temperature.

### Refinement

H atoms bound to C atoms were placed in calculated positions and treated as riding on their parent atoms, with C—H = 0.97 Å (methylene), C—H = 0.93 Å (aromatic) and with U_iso_(H) = 1.2U_eq_(C). H atoms O atoms were initially located in a difference Fourier map and treated as riding on their parent atoms, with O—H = 0.85 Å, U_iso_(H) = 1.5 U_eq_(N/O).

### Crystal data

**Table:**

C_10_H_8_N_2_·C_10_H_10_O_6_·H_2_O	*F*(000) = 840
*M**_r_* = 400.38	*D*_x_ = 1.408 Mg m^−^^3^
Monoclinic, *P*2_1_/*c*	Mo *K*α radiation, λ = 0.71073 Å
Hall symbol: -P 2ybc	Cell parameters from 11161 reflections
*a* = 11.566 (5) Å	θ = 3.0–27.5°
*b* = 9.712 (5) Å	µ = 0.11 mm^−^^1^
*c* = 16.810 (7) Å	*T* = 291 K
β = 90.81 (2)°	Rod, colorless
*V* = 1888.2 (15) Å^3^	0.20 × 0.18 × 0.12 mm
*Z* = 4	

### Data collection

**Table:**

Rigaku R-AXIS RAPID diffractometer	4306 independent reflections
Radiation source: fine-focus sealed tube	2654 reflections with *I* > 2σ(*I*)
graphite	*R*_int_ = 0.030
ω scan	θ_max_ = 27.5°, θ_min_ = 3.0°
Absorption correction: multi-scan (*ABSCOR*; Higashi, 1995)	*h* = −15→14
*T*_min_ = 0.979, *T*_max_ = 0.987	*k* = −12→12
18105 measured reflections	*l* = −21→21

### Refinement

**Table:**

Refinement on *F*^2^	Primary atom site location: structure-invariant direct methods
Least-squares matrix: full	Secondary atom site location: difference Fourier map
*R*[*F*^2^ > 2σ(*F*^2^)] = 0.039	Hydrogen site location: inferred from neighbouring sites
*wR*(*F*^2^) = 0.113	H-atom parameters constrained
*S* = 0.97	*w* = 1/[σ^2^(*F*_o_^2^) + (0.0647*P*)^2^] where *P* = (*F*_o_^2^ + 2*F*_c_^2^)/3
4306 reflections	(Δ/σ)_max_ < 0.001
262 parameters	Δρ_max_ = 0.17 e Å^−^^3^
0 restraints	Δρ_min_ = −0.15 e Å^−^^3^

### Special details

**Table:**

Geometry. All esds (except the esd in the dihedral angle between two l.s. planes) are estimated using the full covariance matrix. The cell esds are taken into account individually in the estimation of esds in distances, angles and torsion angles; correlations between esds in cell parameters are only used when they are defined by crystal symmetry. An approximate (isotropic) treatment of cell esds is used for estimating esds involving l.s. planes.
Refinement. Refinement of *F*^2^ against ALL reflections. The weighted *R*-factor wR and goodness of fit *S* are based on *F*^2^, conventional *R*-factors *R* are based on *F*, with *F* set to zero for negative *F*^2^. The threshold expression of *F*^2^ > σ(*F*^2^) is used only for calculating *R*-factors(gt) etc. and is not relevant to the choice of reflections for refinement. *R*-factors based on *F*^2^ are statistically about twice as large as those based on *F*, and *R*- factors based on ALL data will be even larger.

### Fractional atomic coordinates and isotropic or equivalent isotropic displacement parameters (Å^2^)

**Table:**

	*x*	*y*	*z*	*U*_iso_*/*U*_eq_	
N1	0.60888 (10)	0.06435 (13)	0.44070 (7)	0.0574 (3)	
C1	0.59235 (13)	−0.04189 (17)	0.39244 (9)	0.0616 (4)	
H1	0.5467	−0.0293	0.3470	0.074*	
C2	0.63933 (12)	−0.16976 (16)	0.40627 (8)	0.0588 (4)	
H2	0.6265	−0.2406	0.3700	0.071*	
C3	0.70588 (11)	−0.19349 (14)	0.47429 (8)	0.0462 (3)	
C4	0.72238 (12)	−0.08127 (15)	0.52468 (9)	0.0574 (4)	
H4	0.7662	−0.0910	0.5712	0.069*	
C5	0.67402 (13)	0.04364 (16)	0.50574 (9)	0.0613 (4)	
H5	0.6873	0.1175	0.5399	0.074*	
C6	0.75714 (11)	−0.33068 (14)	0.49263 (8)	0.0460 (3)	
C7	0.72463 (14)	−0.44768 (16)	0.45093 (9)	0.0662 (4)	
H7	0.6697	−0.4416	0.4101	0.079*	
C8	0.77316 (14)	−0.57246 (17)	0.46966 (10)	0.0672 (4)	
H8	0.7498	−0.6493	0.4406	0.081*	
C9	0.88302 (13)	−0.47824 (16)	0.56821 (9)	0.0609 (4)	
H9	0.9375	−0.4879	0.6091	0.073*	
C10	0.83819 (12)	−0.34962 (15)	0.55282 (9)	0.0579 (4)	
H10	0.8626	−0.2748	0.5832	0.069*	
C11	0.27722 (11)	0.59624 (14)	0.26783 (7)	0.0458 (3)	
C12	0.32396 (13)	0.71838 (15)	0.29545 (8)	0.0554 (4)	
H12	0.3895	0.7167	0.3282	0.066*	
C13	0.27396 (13)	0.84376 (16)	0.27477 (9)	0.0597 (4)	
H13	0.3061	0.9256	0.2935	0.072*	
C14	0.17787 (14)	0.84657 (15)	0.22704 (9)	0.0594 (4)	
H14	0.1442	0.9305	0.2135	0.071*	
C15	0.12991 (12)	0.72505 (14)	0.19859 (9)	0.0561 (4)	
H15	0.0644	0.7282	0.1659	0.067*	
C16	0.17855 (11)	0.59898 (13)	0.21839 (8)	0.0460 (3)	
C17	0.04541 (11)	0.47710 (14)	0.13642 (8)	0.0495 (3)	
H17A	0.0694	0.5276	0.0897	0.059*	
H17B	−0.0208	0.5239	0.1587	0.059*	
C18	0.01249 (11)	0.33352 (14)	0.11347 (8)	0.0479 (3)	
C19	0.41863 (12)	0.46231 (15)	0.33609 (8)	0.0542 (4)	
H19A	0.4001	0.5044	0.3867	0.065*	
H19B	0.4818	0.5135	0.3130	0.065*	
C20	0.45447 (12)	0.31491 (16)	0.34906 (9)	0.0548 (4)	
N2	0.85154 (10)	−0.58951 (13)	0.52696 (7)	0.0564 (3)	
O1	0.13736 (8)	0.47386 (9)	0.19334 (6)	0.0545 (3)	
O2	0.05129 (9)	0.23016 (10)	0.14397 (6)	0.0597 (3)	
O3	−0.06491 (9)	0.33379 (10)	0.05595 (6)	0.0651 (3)	
H23	−0.0867	0.2512	0.0481	0.098*	
O4	0.32100 (8)	0.46709 (9)	0.28462 (6)	0.0531 (3)	
O5	0.42135 (9)	0.21927 (11)	0.30849 (7)	0.0710 (3)	
O6	0.52702 (9)	0.30628 (11)	0.40864 (6)	0.0709 (3)	
H24	0.5506	0.2251	0.4185	0.106*	
O7	0.17765 (9)	0.17334 (11)	0.28219 (6)	0.0712 (3)	
H21	0.2472	0.2012	0.2804	0.107*	
H22	0.1466	0.2093	0.2410	0.107*	

### Atomic displacement parameters (Å^2^)

**Table:**

	*U*^11^	*U*^22^	*U*^33^	*U*^12^	*U*^13^	*U*^23^
N1	0.0485 (7)	0.0614 (8)	0.0619 (7)	0.0020 (6)	−0.0057 (6)	0.0183 (6)
C1	0.0563 (9)	0.0752 (11)	0.0530 (9)	0.0049 (8)	−0.0126 (7)	0.0131 (8)
C2	0.0600 (9)	0.0681 (10)	0.0477 (8)	0.0021 (7)	−0.0126 (7)	0.0027 (7)
C3	0.0390 (7)	0.0545 (8)	0.0450 (7)	−0.0045 (6)	−0.0039 (6)	0.0072 (6)
C4	0.0596 (9)	0.0545 (9)	0.0576 (9)	−0.0024 (7)	−0.0206 (7)	0.0064 (7)
C5	0.0631 (9)	0.0544 (9)	0.0659 (10)	−0.0012 (7)	−0.0148 (8)	0.0071 (7)
C6	0.0416 (7)	0.0524 (8)	0.0438 (7)	−0.0029 (6)	−0.0028 (6)	0.0038 (6)
C7	0.0703 (10)	0.0606 (10)	0.0669 (10)	0.0008 (8)	−0.0296 (8)	−0.0013 (7)
C8	0.0746 (11)	0.0555 (9)	0.0707 (10)	−0.0005 (8)	−0.0216 (9)	−0.0062 (8)
C9	0.0601 (9)	0.0614 (10)	0.0605 (9)	0.0063 (7)	−0.0208 (7)	−0.0002 (7)
C10	0.0591 (8)	0.0562 (9)	0.0578 (9)	0.0015 (7)	−0.0184 (7)	−0.0038 (7)
C11	0.0460 (7)	0.0424 (7)	0.0491 (8)	0.0020 (6)	−0.0019 (6)	0.0038 (6)
C12	0.0569 (8)	0.0524 (8)	0.0564 (9)	−0.0040 (7)	−0.0123 (7)	−0.0008 (7)
C13	0.0711 (10)	0.0460 (8)	0.0619 (9)	−0.0055 (7)	−0.0024 (8)	−0.0063 (7)
C14	0.0701 (9)	0.0429 (8)	0.0653 (9)	0.0085 (7)	−0.0010 (8)	−0.0015 (7)
C15	0.0536 (8)	0.0473 (8)	0.0673 (9)	0.0062 (6)	−0.0098 (7)	−0.0008 (7)
C16	0.0434 (7)	0.0412 (7)	0.0532 (8)	0.0010 (6)	−0.0036 (6)	−0.0006 (6)
C17	0.0436 (7)	0.0486 (8)	0.0559 (8)	0.0043 (6)	−0.0107 (6)	0.0010 (6)
C18	0.0419 (7)	0.0512 (8)	0.0504 (8)	0.0016 (6)	−0.0037 (6)	0.0006 (6)
C19	0.0486 (8)	0.0563 (9)	0.0572 (8)	−0.0028 (6)	−0.0138 (7)	0.0076 (7)
C20	0.0420 (7)	0.0605 (9)	0.0615 (9)	−0.0020 (7)	−0.0072 (7)	0.0113 (7)
N2	0.0542 (7)	0.0556 (7)	0.0592 (7)	0.0048 (6)	−0.0051 (6)	0.0036 (6)
O1	0.0508 (5)	0.0404 (5)	0.0718 (6)	0.0025 (4)	−0.0210 (5)	−0.0003 (4)
O2	0.0639 (6)	0.0475 (6)	0.0672 (7)	0.0036 (5)	−0.0183 (5)	0.0005 (5)
O3	0.0661 (6)	0.0540 (6)	0.0743 (7)	−0.0046 (5)	−0.0293 (6)	0.0030 (5)
O4	0.0492 (5)	0.0462 (6)	0.0635 (6)	0.0002 (4)	−0.0167 (4)	0.0052 (4)
O5	0.0635 (7)	0.0599 (7)	0.0888 (8)	0.0060 (5)	−0.0230 (6)	−0.0003 (6)
O6	0.0665 (7)	0.0639 (7)	0.0812 (8)	0.0023 (5)	−0.0319 (6)	0.0154 (6)
O7	0.0797 (7)	0.0581 (7)	0.0750 (7)	−0.0155 (5)	−0.0260 (6)	0.0135 (5)

### Geometric parameters (Å, °)

**Table:**

N1—C1	1.325 (2)	C12—H12	0.9300
N1—C5	1.3342 (18)	C13—C14	1.362 (2)
C1—C2	1.374 (2)	C13—H13	0.9300
C1—H1	0.9300	C14—C15	1.386 (2)
C2—C3	1.3885 (18)	C14—H14	0.9300
C2—H2	0.9300	C15—C16	1.3861 (19)
C3—C4	1.392 (2)	C15—H15	0.9300
C3—C6	1.489 (2)	C16—O1	1.3695 (16)
C4—C5	1.371 (2)	C17—O1	1.4208 (15)
C4—H4	0.9300	C17—C18	1.495 (2)
C5—H5	0.9300	C17—H17A	0.9700
C6—C10	1.3818 (18)	C17—H17B	0.9700
C6—C7	1.384 (2)	C18—O2	1.2105 (16)
C7—C8	1.370 (2)	C18—O3	1.3083 (16)
C7—H7	0.9300	C19—O4	1.4136 (15)
C8—N2	1.3237 (18)	C19—C20	1.505 (2)
C8—H8	0.9300	C19—H19A	0.9700
C9—N2	1.3320 (19)	C19—H19B	0.9700
C9—C10	1.376 (2)	C20—O5	1.2113 (18)
C9—H9	0.9300	C20—O6	1.3002 (16)
C10—H10	0.9300	O3—H23	0.8499
C11—O4	1.3803 (17)	O6—H24	0.8500
C11—C12	1.3812 (19)	O7—H21	0.8499
C11—C16	1.4025 (18)	O7—H22	0.8501
C12—C13	1.390 (2)		
			
C1—N1—C5	117.34 (13)	C14—C13—C12	119.87 (13)
N1—C1—C2	123.17 (12)	C14—C13—H13	120.1
N1—C1—H1	118.4	C12—C13—H13	120.1
C2—C1—H1	118.4	C13—C14—C15	120.36 (13)
C1—C2—C3	120.17 (14)	C13—C14—H14	119.8
C1—C2—H2	119.9	C15—C14—H14	119.8
C3—C2—H2	119.9	C16—C15—C14	120.67 (13)
C2—C3—C4	116.16 (13)	C16—C15—H15	119.7
C2—C3—C6	122.24 (13)	C14—C15—H15	119.7
C4—C3—C6	121.60 (11)	O1—C16—C15	124.87 (11)
C5—C4—C3	119.96 (12)	O1—C16—C11	116.23 (11)
C5—C4—H4	120.0	C15—C16—C11	118.90 (12)
C3—C4—H4	120.0	O1—C17—C18	109.80 (10)
N1—C5—C4	123.18 (14)	O1—C17—H17A	109.7
N1—C5—H5	118.4	C18—C17—H17A	109.7
C4—C5—H5	118.4	O1—C17—H17B	109.7
C10—C6—C7	115.98 (13)	C18—C17—H17B	109.7
C10—C6—C3	122.33 (12)	H17A—C17—H17B	108.2
C7—C6—C3	121.68 (12)	O2—C18—O3	124.09 (13)
C8—C7—C6	120.14 (13)	O2—C18—C17	124.94 (12)
C8—C7—H7	119.9	O3—C18—C17	110.98 (11)
C6—C7—H7	119.9	O4—C19—C20	109.62 (11)
N2—C8—C7	123.49 (14)	O4—C19—H19A	109.7
N2—C8—H8	118.3	C20—C19—H19A	109.7
C7—C8—H8	118.3	O4—C19—H19B	109.7
N2—C9—C10	122.64 (12)	C20—C19—H19B	109.7
N2—C9—H9	118.7	H19A—C19—H19B	108.2
C10—C9—H9	118.7	O5—C20—O6	125.43 (14)
C9—C10—C6	120.57 (13)	O5—C20—C19	124.30 (12)
C9—C10—H10	119.7	O6—C20—C19	110.26 (13)
C6—C10—H10	119.7	C8—N2—C9	117.18 (13)
O4—C11—C12	124.82 (11)	C16—O1—C17	116.18 (10)
O4—C11—C16	115.56 (11)	C18—O3—H23	108.1
C12—C11—C16	119.61 (11)	C11—O4—C19	116.26 (10)
C11—C12—C13	120.59 (12)	C20—O6—H24	114.3
C11—C12—H12	119.7	H21—O7—H22	103.2
C13—C12—H12	119.7		

### Hydrogen-bond geometry (Å, °)

**Table:**

*D*—H···*A*	*D*—H	H···*A*	*D*···*A*	*D*—H···*A*
O3—H23···N2^i^	0.85	1.76	2.6051 (19)	173
O6—H24···N1	0.85	1.74	2.5871 (19)	176
O7—H21···O5	0.85	2.07	2.8817 (19)	160
O7—H22···O2	0.85	1.97	2.7826 (17)	161

Symmetry codes: (i) *x*−1, −*y*−1/2, *z*−1/2.
